# Psychometric properties of the de Morton Mobility Index in ICU patients

**DOI:** 10.1186/cc14555

**Published:** 2015-03-16

**Authors:** J Sommers, T Vredeveld, J Horn, RH Engelbert, R Lindeboom, M Vd Schaaf

**Affiliations:** 1 Academical Medical Center, Amsterdam, the Netherlands; 2 Amsterdam School of Health Professions (ASHP), University of Applied Sciences, Amsterdam, the Netherlands

## Introduction

Many ICU patients develop ICU-acquired muscle weakness (ICU-AW) due to inactivity and critical illness. ICU-AW is associated with short-term and long-term physical impairments and impaired functional status [1]. The de Morton Mobility Index (DEMMI) was developed to measure changes in mobility across clinical settings and proved to be reliable, feasible and sensitive to small but clinically relevant changes in functioning [2]. Our aim was to evaluate the psychometric properties of the DEMMI in ICU patients.

## Methods

The inter-rater reliability and validity were determined in a prospective observational study. Patients were included and assessed by two independent raters until hospital discharge. Reliability was expressed using the intraclass correlations (ICC). To evaluate the validity, the DEMMI scores were compared with the Barthel Index (BI), Katz-ADL and manual muscle testing (MMT).

## Results

A total of 115 ICU patients were included. The average age was 61 years and 67% of the patients were male. ICU admission diagnoses were 53% acute surgery, 14% elective surgery and 33% were admitted for medical nonsurgical reasons. Inter-rater reliability of the DEMMI was high: intraclass correlation coefficient (ICC) ranging from >0.91 (range 0.85 to 0.94) at ICU admission, >0.98 (range 0.96 to 0.99) at the MICU and >0.97 (range 0.96 to 0.98) at the general ward. Internal consistency reliabilities (Cronbach α) of the DEMMI were 0.84, 0.87 and 0.98 at the ICU, MICU and hospital ward respectively. Validity coefficients (Spearman's rank correlations) with BI, Katz-ADL and MMT were 0.63, -0.45 and 0.62.

## Conclusion

The DEMMI is a reliable, responsive and feasible measurement instrument for the assessment of mobility in critically ill ICU patients.
